# Effect of Laser Surface Texturing and Fabrication Methods on Tribological Properties of Ti6Al4V/HAp Biocomposites

**DOI:** 10.3390/ma18112468

**Published:** 2025-05-24

**Authors:** Julia Sadlik, Edyta Kosińska, Agnieszka Tomala, Magdalena Bańkosz, Marko Polajnar, Rahul Kumar, Mitjan Kalin, Gaia Kravanja, Luka Hribar, Irina Hussainova, Marek Nykiel, Agnieszka Sobczak-Kupiec, Josef Jampilek

**Affiliations:** 1 Department of Materials Science, Faculty of Materials Engineering and Physics, CUT Doctoral School, Cracow University of Technology, 37 Jana Pawła II Av., 31-864 Krakow, Poland; edyta.kosinska@doktorant.pk.edu.pl (E.K.); magdalena.bankosz@pk.edu.pl (M.B.); 2 Department of Materials Science, Faculty of Materials Engineering and Physics, Cracow University of Technology, 37 Jana Pawła II Av., 31-864 Krakow, Poland; agnieszka.tomala@pk.edu.pl (A.T.); marek.nykiel@pk.edu.pl (M.N.); agnieszka.sobczak-kupiec@pk.edu.pl (A.S.-K.); 3 Laboratory for Tribology and Interface Nanotechnology, Faculty of Mechanical Engineering, University of Ljubljana, Bogišićeva 8, 1000 Ljubljana, Slovenia; marko.polajnar@fs.uni-lj.si (M.P.); rahul.kumar@fs.uni-lj.si (R.K.); mitjan.kalin@fs.uni-lj.si (M.K.); 4 Department of Mechanical and Industrial Engineering, Tallinn University of Technology, 19086 Tallinn, Estonia; irina.hussainova@taltech.ee; 5 Laboratory for Laser Techniques, Faculty of Mechanical Engineering, University of Ljubljana, Aškerčeva 6, 1000 Ljubljana, Slovenia; gaia.kravanja@fs.uni-lj.si (G.K.); luka.hribar@fs.uni-lj.si (L.H.); 6 Department of Analytical Chemistry, Faculty of Natural Sciences, Comenius University, Ilkovicova 6, 842 15 Bratislava, Slovakia; 7 Department of Chemical Biology, Faculty of Science, Palacky University Olomouc, Slechtitelu 27, 779 00 Olomouc, Czech Republic

**Keywords:** titanium alloy, hydroxyapatite, laser surface texturing, SPS, biocomposite, friction, biomaterial

## Abstract

Bone diseases lead to an increasing demand for implants to treat long bone defects and for load-bearing applications. Osteoporosis care and accidental injuries are major contributors to this rising need. Our research aims to demonstrate innovative material systems and methods for preparing implants that can be used in regenerative medicine. We hypothesize that by combining titanium alloys (Ti6Al4V) with hydroxyapatite (Hap), we can enhance biocompatibility and tribo-mechanical performance, which are critical for the longevity of Ti-based surgical implants. Additionally, we investigate the application of laser surface treatments to expose the underlying porosity, thereby enhancing cell transport and promoting cell growth. In this study, we investigate the effects of two fabrication techniques—Spark Plasma Sintering (SPS) and powder metallurgy (PM)—on the properties of laser-textured Ti64/Hap biocomposites. Our findings demonstrate that the selected processing route significantly influences the microstructure, tribological performance, and surface properties of these materials. An X-ray diffraction (XRD) analysis corroborates our results from incubation studies in simulated body fluids, highlighting the impact of phase transformations during sintering on the chemical properties of Ti-Hap composites. Additionally, while laser surface texturing was found to slightly increase the friction coefficient, it markedly enhanced the wear resistance, particularly for the PM and SPS Ti + 5%Hap composites.

## 1. Introduction

Titanium alloy/hydroxyapatite (Ti/Hap) composites have become a focal point in biomedical applications, especially in bone implants thanks to their advantageous mechanical properties and bioactivity. The combination of hydroxyapatite, which closely mimics the mineral component of bone, with titanium, known for its strength and durability, results in materials that can effectively promote osseointegration and support bone regeneration [1,2]. The hypothesis of this research states that combining a Ti alloy with hydroxyapatite (Hap) and surface laser texturing leads to the obtainment of an excellent biomaterial supporting bone growth and eliminating the problem of the loosening of the implant by its integration with bone. The laser surface treatment enhances the underlying porosity, improving the cell transport and cell growth of the biomaterial [3].

Various methods have been developed for synthesizing Ti/Hap composites, each with distinct advantages and limitations. Many techniques enable covering or reinforcing on the implant surface with Hap films, such as electrophoresis (EPD), sol–gel processes, thermal sprays, dusting plasma sprayed deposition, hot isostatic pressing, dip coating, pulsed laser deposition (PLD), and ion-beam-assisted deposition (IBAD). One prevalent technique is plasma spraying, which allows for the deposition of hydroxyapatite coatings onto titanium substrates. This method enhances the osteoconductivity of the implants, facilitating better integration with the bone tissue [1,2]. Additionally, thermal spraying and sputter coating have also been employed to achieve similar outcomes [1,4]. These coating techniques are crucial as they create a bioactive surface that encourages cell attachment and proliferation, thereby improving the overall performance of the implants [5]. The main limitation of deposited Hap coatings is the poor adhesion between the ceramic coating and the metal surface, causing gradual delamination [6].

Another approach involves the use of powder metallurgy, where titanium alloy and hydroxyapatite powders are mixed and sintered to form a composite material. This method allows for better control over the microstructure and the composition of the final product, resulting in improved mechanical properties and bioactivity [7]. For instance, studies have shown that composites with up to 10% hydroxyapatite exhibit an enhanced density and strength, making them suitable for load-bearing applications [8]. Furthermore, pressureless sintering at lower temperatures has been explored to mitigate the decomposition of hydroxyapatite, which can occur at higher temperatures, thus preserving its bioactive properties [9]. In addition, the pressureless sintering of Hap can achieve a near-theoretical density between 1000 °C and 1200 °C; however, this process is limited by its significant grain growth and potential decomposition, as Hap becomes unstable at temperatures exceeding 1300 °C [10,11]. Sintering at higher temperatures, particularly above 1300 °C, results in the decomposition of Hap into tricalcium phosphate (TCP) and anhydrous calcium phosphates, which affects the material’s dissolution rates in physiological conditions. This necessitates the careful selection of the processing method.

Spark Plasma Sintering (SPS) has shown promise for sintering Ti-Hap composites, offering control over densification while preserving Hap’s bioactivity. Unlike conventional sintering (such as pressureless sintering), SPS minimizes the Ti-induced Hap decomposition by enabling ultra-fast heating and short dwell times, suppressing unwanted phase transformations, like CaO and TCP formation. The rapid densification process enhances the interfacial bonding between Ti and Hap, refining the composite microstructure with a reduced porosity and uniform phase distribution [12]. By preventing excessive grain growth and degradation, SPS ensures Ti-Hap composites with superior mechanical integrity and biocompatibility. For the optimal SPS of titanium-Ti-Hap composites, sintering at temperatures between 950 °C and 1100 °C, under a pressure of approximately 30–50 Mpa, and with a dwell time of 5 to 8 min, has been shown to achieve a near-theoretical density and superior mechanical properties [13].

Kumar et al. [14] demonstrated that SPS at 950 °C enables the fabrication of dense Hap-Ti composites (5, 10, and 20 wt%) with an enhanced fracture toughness (4–5 Mpa·m1/2). Among the compositions, Hap-10Ti was identified as optimal, offering a good hardness value (~6.1 ± 0.6 Gpa), the highest fracture toughness (~4.6 Mpa·m1/2), and a ~35% improvement over pure Hap (~3.4 Mpa·m1/2). The fracture toughness of Hap bioceramics does not exceed ~1.2 Mpa·m1/2 [15], where natural human bone has a toughness of 2–12 Mpa·m1/2 [13,16]. It is reported that an increasing porosity results in a decline in mechanical strength, with strength decreasing exponentially as the porosity increases [14]. Therefore, a careful selection of the sintering process is essential to achieving Ti-Hap composites with optimal mechanical properties.

The laser surface texturing of Ti alloys has been extensively used in studies emphasizing its tribological [17,18,19,20,21,22,23,24,25] and bio-tribological performance [26,27,28].

What is more, the shape and the size of the textures varied between channels and grooves [18,19,28,29], pillars [27], crosshatches [20,21], and, most commonly, dimples [17,22,23,24,25,26]. Furthermore, a significant variation in the size and the depth of the textures between these studies was also found [3]. Namely, the textured features varied from smaller textures with diameter below 100 µm [25,28,29] to larger ones with a diameter up to 300 µm [17,21,22,23,24,26]. The same observation was also found for the depth of the textures that were, in some studies, around 10 µm or smaller [24,26,28,29], and in some studies they were in the range of 15–40 µm [17,22,23,25] and were even up to 125 µm deep [21]. These differences in surface texture may originate from the tribological testing conditions, which is another important aspect that should be taken into account.

Namely, the tribological studies of the Ti alloy commonly used low sliding speeds up to 100 mm/s [18,26,30,31,32,33,34,35,36,37,38,39,40,41] with strokes up to a few millimeters [34,42,43] and low normal loads up to several N [18,19,28,30,31,32,33,34,35,42,43,44,45,46,47]. High loads up to 100 N were used only in specialized studies using conformal contacts [39] or fretting conditions [48]. Despite the low loads and lubricated conditions with PBS or SBF solutions [19,27,28,29,31,32,34,40,42,47,49,50], the contact pressures reached in these contacts were high—in an order of magnitude of a few 100 Mpa [18,32,39,43,44,48]. This was due to the use of concentrated ball-on-disk point contacts and ceramic balls (Al_2_O_3_, ZrO_2_, WC, or Si_3_N_4_) as a counter surface [26,28,30,32,34,36,38,41,42,46,47,48,49,50]. The contact pressures were thus much higher compared to those in real applications. For example, the contact pressure in the human hip joint is usually around 10 Mpa. However, higher pressures and hard ceramic counter bodies in tribological studies are used in order to induce the measurable wear only on the Ti alloy and in the reasonable time of the tribological test.

Despite many tribological studies of Ti alloys, only a few of them studied the Ti-HAP composites without laser surface texturing [51,52,53,54].

Our research aims to address the critical need for implants designed to treat long bone defects and support load-bearing applications by exploring innovative material systems and surface modification methods for implants that can be utilized in regenerative medicine. Our study focuses on the comparative analysis of two fabrication techniques—Spark Plasma Sintering (SPS) and powder metallurgy (PM)—on the properties of laser-textured Ti6Al4V alloy/hydroxyapatite (Hap) composites. The results reveal that the chosen processing route plays a significant role in determining the microstructure, tribological performance, and surface properties of the materials. X-ray fluorescence and X-ray diffraction (XRD) analyses have supported our findings derived from incubation studies conducted in simulated body fluids. This analysis highlights the critical impact of phase transformations during the sintering process on the chemical properties of Ti/Hap composites. Furthermore, our results indicate that while laser surface texturing slightly increases the friction coefficient, it substantially enhances wear resistance, particularly in the PM and SPS Ti + 5%Hap composites.

These findings underscore the importance of optimizing both material composition and processing techniques to develop effective implants for the treatment of bone diseases and the repair of long bone defects.

## 2. Materials and Methods

### 2.1. Materials

#### 2.1.1. Powder Metallurgy Samples

The materials were made using synthesized hydroxyapatite, carboxymethylcellulose (Car), Natriumsalz, M.W. ca.250000 (CAS: 900-32-4), and the titanium alloy (Ti64) Ti-6Al-4V, grade 5, with a particle size range of 15–53 µm, which was purchased from AMC Powders Co., Ltd. (Beijing, China).

Hydroxyapatite was synthesized using the acid–base method, which is described in our other article [55].

#### 2.1.2. Spark Plasma Sintering Feedstock

Gas-atomized Ti6Al4V powder (Table 1) with a particle size distribution of 20–63 µm was sourced from SLM Solutions. Hydroxyapatite (Hap) powder, with a particle size of less than 10 µm and a purity exceeding 98%, was synthesized in-house, following the procedure detailed in our previous study [56], i.e., the Hap powder was synthesized with the help of wet precipitation through dissolving the appropriate amount CaCO_3_ in demineralized water. Urea phosphate (UPH, CO(NH_2_)_2_–H_3_PO_4_, Sigma-Aldrich, St. Louis, MO, USA) was added into the solution to gain Ca/P with a molar ratio of 1.67, which was required for the stoichiometric Hap. Continuous stirring was followed by 12 h of aging at room temperature.

### 2.2. Preparing Ti/Hap Samples

#### 2.2.1. Powder Metallurgy Samples

The first step in the preparation of the materials was to prepare powders with a specific hydroxyapatite content and a porophore (Car). The next step was cold isostatic pressing, which made it possible to obtain samples with a diameter of 13 mm and a height of 4 mm. Material preparation procedures are described in our other article [55]. The compositions of the samples produced are shown in Table 2.

#### 2.2.2. Sintering PM Samples

The last step for the powder metallurgy samples was sintering. The high-temperature sintering process of Ti6Al4V samples with varying Hap and CMC contents was conducted using a Nabertherm furnace. The samples were heated at 2°/min to a temperature of 1200 degrees under argon gas protection. The introduction of argon gas was intended to reduce the evaporation of the samples and also to limit the access of oxygen, which causes oxidation. The holding time at this temperature was 4 h. After this time, the samples were cooled to room temperature at a rate of 2°/min. The materials were sintered in a quartz tube, which provided a better seal than a ceramic tube. The samples were placed in alumina crucibles and backfilled with zirconia powder to provide additional protection against the oxidation of the titanium alloy. Before sintering, the materials were placed in a laboratory dryer at 300 °C for 1h in order to evaporate the organic components, which come from the Car additive.

#### 2.2.3. Spark Plasma Sintering

To prepare Ti-HA composite powders, 5 and 10 vol% Hap was blended with Ti6Al4V using a planetary ball milling process. The mixing was conducted for 2 h in a plastic jar utilizing yttria-stabilized zirconia (YSZ) milling media (10 balls, 5 mm diameter).

The consolidated Ti-HA composite bulks were fabricated using a Spark Plasma Sintering (SPS) system, the KCE^®^-FCT HP D 10-GB (FCT Systeme GmbH, Frankenblick, Germany). Approximately 6 g of the mixed powder was loaded into a 20 mm diameter graphite mold without pre-compaction. Graphite foils were placed at both ends of the mold to mitigate adhesion between the powder and the die surfaces. A graphite spacer was also incorporated to facilitate sample removal post-sintering. The sintering process was conducted at 1100 °C, with a heating rate of ~100 °C/min, under a constant uniaxial pressure of 50 Mpa. A dwell time of 5 min was maintained, and the entire process was performed under vacuum conditions. Direct current (DC) pulses were applied intermittently with an on–off pulse ratio of 12:2, ensuring uniform heating and particle surface activation. The instantaneous pulsed DC discharge, delivered through the graphite punches, facilitated surface purification and promoted localized self-heating, leading to rapid and efficient densification as reported elsewhere [57]. Temperature monitoring during SPS was carried out using a K-type thermocouple. The final sintered specimens were consolidated into cylindrical pellets (20 mm diameter and 5 mm height) with ~99% relative density. The surfaces of the composite bulks were then polished to achieve a surface roughness (Ra) of 0.2 µm, ensuring optimal conditions for subsequent characterization. The compositions of the samples produced are shown in Table 3. After SPS, the sintered cylindrical samples were polished to a surface roughness of Ra 0.2 ± 0.02 μm using a Phoenix 4000 (Buehler, Lake Bluff, IL, USA) system and subsequently cleaned with acetone and ethylene alcohol. The bulk density of the composites was determined using Archimedes’ principle, with distilled water as the immersion medium, employing a Mettler Toledo ME204 balance (Mettler Toledo, Columbus, OH, USA) with an accuracy of 0.1 mg. The bulk Vickers hardness (HV30 and HV0.2) was measured using an Indentec 5030 SKV unit (Stourbridge, West Midlands, UK), applying indentation loads of 30 kg and 0.2 kg for 10 s. The reported values represent the mean of at least three independent measurements.

### 2.3. Scanning Electron Microscopy with Elemental Composition Analysis

The surface and structure of the produced materials can be observed and analyzed by examining the material with a scanning electron microscope (SEM). The samples were analyzed after the sintering process to visualize their surface morphology. The samples were dried and then sputtered shortly before measurement. Observations were performed using a JEOL IT200 (JEOL Ltd., Peabody, MA, USA). Prior to measurement, the samples were prepared by sputtering with a gold nanolayer. This was carried out using a Smart Coater DII-29030SCTR (Joel Ltd., Peabody, MA, USA) auto-vacuum sputtering machine.

### 2.4. Density and Porosity of Samples

The apparent density of the materials produced was determined by applying the Archimedes method (hydrostatic weighing). The first step was to weigh the materials in air and then in distilled water. In order to determine the density of the distilled water, its temperature was determined. Then, the density of the sample was calculated using the following formula:

(1) $$\rho =\frac{m_{1}}{m_{1}-m_{2}}\cdot \rho_{water}$$

m_1_—the weight of the dry sample;

m_2_—the apparent mass of a sample saturated with liquid and weighed in liquid;

*ρ*_*water*_—the density of distilled water at a specific temperature.

From the results obtained, the porosity of the sample was determined using the following formula:

(2) $$\rho =\frac{m_{1}}{m_{3}-m_{2}}\cdot \rho_{water}$$

m_1_—the weight of the dry sample;

m_2_—the apparent mass of a sample saturated with liquid and weighed in liquid;

m_3_—the mass of a 30 min liquid-saturated sample, weighed in air;

*ρ*_*water*_—the density of distilled water at a specific temperature.

### 2.5. X-Ray Fluorescence

An X-ray fluorescence analysis of the samples was carried out to determine the chemical composition of the materials, in particular the content of elements such as titanium, aluminum, vanadium, calcium, phosphorus, and any other additives. The measurement was carried out using an S2 PUMA series II energy-dispersive X-ray fluorescence spectrometer (Bruker, Billerica, MA, USA). The measurement was carried out under room conditions.

### 2.6. XRD Methodology

The phase composition of the sintered Ti-Hap composites was analyzed using X-ray diffraction (XRD) with a PANalytical Aeris diffractometer (Malvern PANalytical, Almelo, The Netherlands). The measurements were conducted in the 9.999–100° 2θ range, with a measurement step of 0.0027° 2θ. A nickel filter, a 13 mm mask, and a 1° gap were used to ensure precise phase identification and detect phase transformations occurring during the sintering process.

### 2.7. Potentiometry and Conductivity Analysis

In order to determine the behavior of the designed material in an environment that stimulates the conditions of a living organism, the material was subjected to incubation tests in an artificial incubation fluid—SBF (simulated body fluid). Each type of material was placed in 80 mL of incubation fluid in a sterile container. Incubation was carried out for a period of 21 days at 36.6 °C in POL-EKO incubator, model ST 5 B SMART (POL-EKO, Wodzisław Śląski, Poland). pH values and conductivity measurements were determined after 1, 3, 7, 10, 12, 14, and 21 days using an Elmetron CX-701 pH-meter (Elmetron, Zabrze, Poland).

### 2.8. Laser Texturing

The laser microdrilling of the samples was performed using an experimental setup, consisting of a nanosecond pulsed fiber laser source (G4 Pulsed Fibre Laser, SPI Lasers UK Ltd., Southampton, UK) with a wavelength of 1070 nm, a maximum average power of 20 W, a pulse duration of 120 ns, and a repetition rate of 55 kHz. The output beam is guided first through an expanding collimating lens and into a scan head (Raylase SS-IIE-10, Raylase GmbH, Weßling, Germany), which deflects the beam along a pre-programmed dotted path over the sample surface, where a single dot represents the drill hole coordinates, and the drilling time (t = 0.054 ms) defines the number of pulses. The coordinates of the dotted array are defined in proprietary software (SamLight, SCAPS GmbH, Oberhaching, Germany), prior to microdrilling. Finally, a telecentric f-theta lens is used to focus the beam (f = 56 mm; Ronar Smith, Singapore).

Textured Ti-HAP samples were analyzed under an optical interferometer to characterize the geometry of the dimples (diameter, depth, and edge) and to check the set raster of the dimple network. Additionally, the digital microscope images of the these samples were recorded.

### 2.9. Tribological Tests

Tribological tests were performed on the multi-functional RTEC—MFT5000 tribometer (RTEC Instruments, San Jose, CA, USA) using a reciprocating sliding module. All titanium alloy/hydroxyapatite disk samples were sliding against a stationary ZrO_2_ ball (diameter of 20 mm) with a frequency of 1 Hz and a set stroke of 2 mm, resulting in a sliding speed of 4 mm/s. The ZrO_2_ ball was loaded against titanium alloy/hydroxyapatite disk samples with 2 N, resulting in 300 Mpa of Hertzian contact pressure. The tests last for 15 min and at least three repetitions were performed for each type of sample. During the tribological tests the Ti-HAP/ZrO_2_ contact pairs were lubricated with 1 mL of PBS (Phosphate Buffer Saline) and the temperature was keep constant at 37 ± 1 °C. The coefficient of friction was measured continuously during the tribological tests, and friction evolutions along with steady-state values are reported in the results.

After the tribological tests the Ti-HAP disk samples were analyzed under an optical interferometer to evaluate the wear resistance properties. Namely, the wear profiles were extracted from the recorded white-light interferometry images and the wear volume was determined. The wear volume was further normalized with an applied normal load (2 N) and the total sliding distance of the test (3.6 m) to obtain the wear coefficient.

## 3. Results

### 3.1. SEM/EDX Analysis

**Figure 1:**
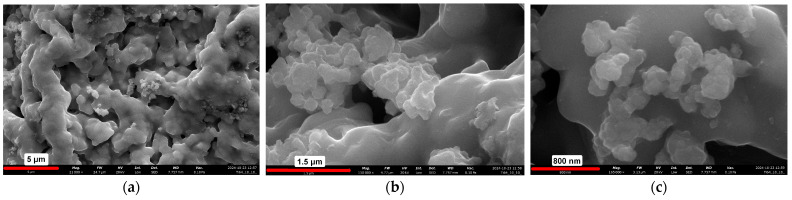
SEM images with rising magnification: 21,000× (**a**), 110,000× (**b**), and 165,000× (**c**) of the corrugated composite surface of the Ti6Al4V alloy with 5% HAP and 5% Car.

The surface morphology of the powder metallurgy samples was observed using scanning electron microscopy (SEM) coupled with energy-dispersive X-ray spectroscopy (EDX). The surface of the Ti6Al4V + 5% HAP and 5% Car composite is depicted in Figure 1. As shown in Figure 1, the surface exhibits significant ridges and corrugations, which create an ideal microenvironment for cell adhesion and growth. Additionally, small cauliflower-like structures, visible in Figure 1b,c, represent hydroxyapatite (Hap) particles interspersed within the Ti6Al4V matrix (Table 4).

**Figure 2:**
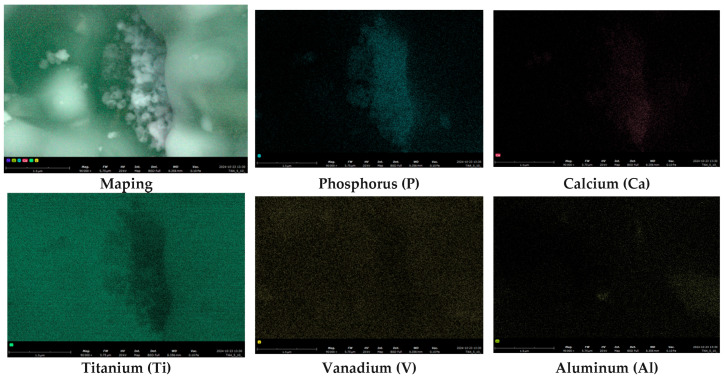
SEM-EDS elemental mapping images of Ti6Al4V alloy with 5% HAP and 5% Car.

Clearly the presence of distinct signals from phosphorus and calcium further confirms the existence of these visible HAp structures (Figure 2).

### 3.2. Porosity Measurements

The porosity and density results for the powder metallurgy samples are shown in Table 5. The values obtained are quite similar and some correlations can be seen. Materials with a higher porophore content adequately achieved a higher porosity. Samples with a Car content of 5% achieved a porosity of approx. 15%, while those with 10% Car achieved a porosity of 22–26%. Consequently, the materials having a higher density are those with less Car addition, as the material is less porous in its volume, while those with an assumed higher porosity similarly have a lower density. It should be noted that the density of the composite relative to the titanium alloy Ti6Al4V itself has also been significantly reduced, as it is 4.3 g/cm^3^, while the hydroxyapatite is around 3.1 g/cm^3^. It should be noted that the assumed addition of porophor does not coincide with the calculated value, but this is also due to the fact that the combustion temperature of the carboxymethylcellulose is on average between 200 and 300 °C which can also result in the removal of material adhering to the Car. And also, the inaccuracy of the method may contribute to why the result is higher than assumed.

### 3.3. XRF Analysis

**Figure 3:**
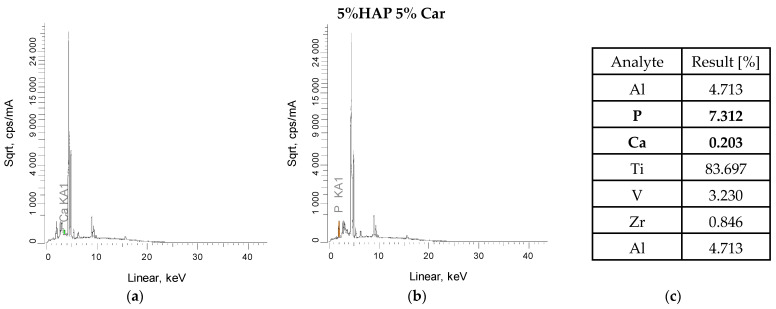

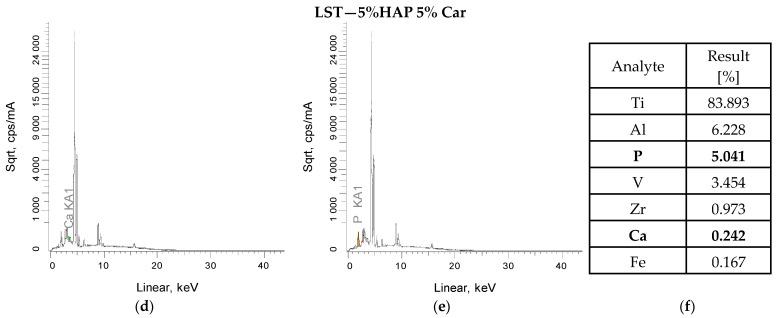
Results of the XRF analysis of the 5% Hap and 5% Car composites before (**a**–**c**) and after laser texturing (**d**–**f**).

**Figure 4:**
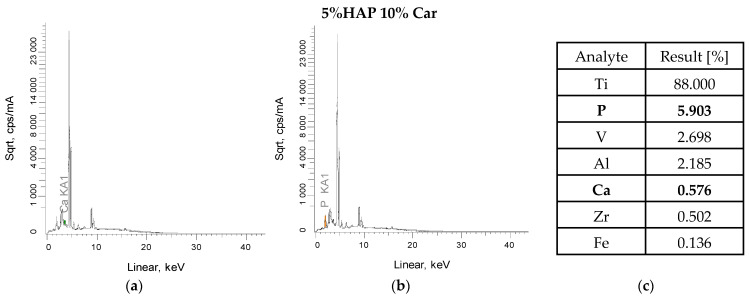

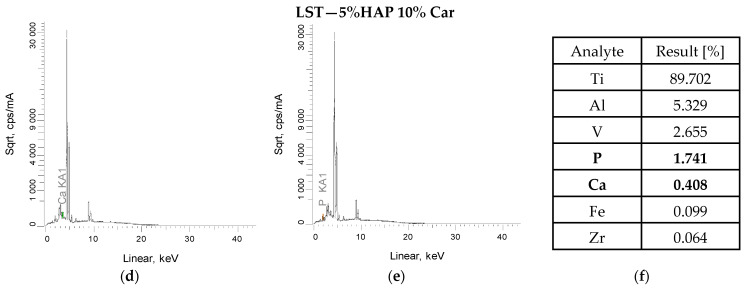
Results of the XRF analysis of the 5% HAp and 10% Car composites before (**a**–**c**) and after laser texturing (**d**–**f**).

XRF measurements were also carried out for the powder metallurgy materials after sintering and after laser structuring (Figure 3 and Figure 4). The tests confirmed the presence of all constituent elements of the titanium alloy, as well as the elements calcium and phosphorus, which confirm the presence of calcium phosphates after the sintering process. The low result is due to the fact that the measurement does not penetrate deeply into the sample, and also any splatter formed during sintering in the protective gas must be borne in mind. Titanium, on the other hand, is the dominant element (approximately 83–89%), which is the main component of this composite. The presence of other elements, such as zirconium, may be due to the technological process and sintering residues, while iron may be due to instrument or apparatus error, but these values are so low that they can be treated as negligible. The main emission lines for the elements that make up hydroxyapatite are Kα (3.69 keV) and Kβ (4.01 keV) for calcium and Kα (2.01 keV), usually with a lower intensity than calcium (Figure 3a,b and Figure 4a,b) All these peaks are seen for the tested materials after sintering. Furthermore, samples after the laser texturing process also show the presence of characteristic calcium and phosphorus peaks (Figure 3d,e and Figure 4d,e).

The results obtained after the laser texturing process do not change significantly. For the 5% H 10% Car sample, the value for titanium before LST was 89%, while after it was 88%. Moreover, during the process itself, and regarding the energy that is generated in contact with the sample, there was no formation or contamination of the materials by other elements. The LST process does not have a major impact on the chemical composition of the materials, nor does it create new structures and elements that affect the sample and its structure.

### 3.4. XRD Analysis Results

**Figure 5:**
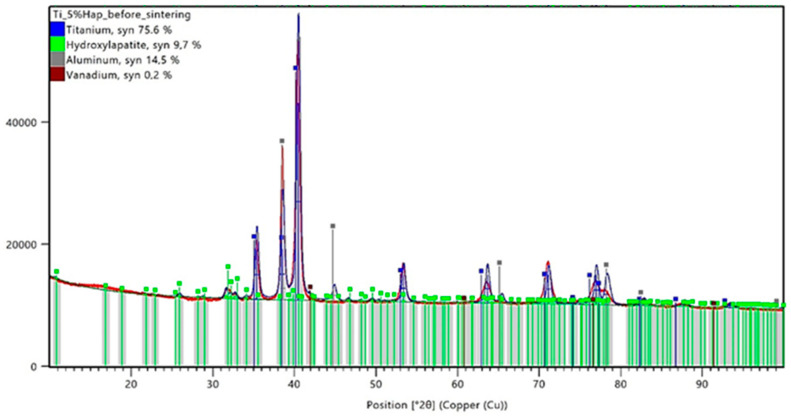
XRD analysis results for a sample containing 5% HAp before the sintering process.

**Figure 6:**
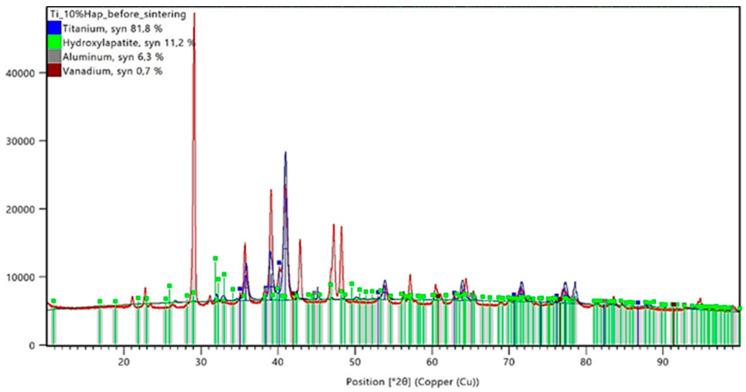
XRD analysis results for a sample containing 10% HAp before the sintering process.

**Figure 7:**
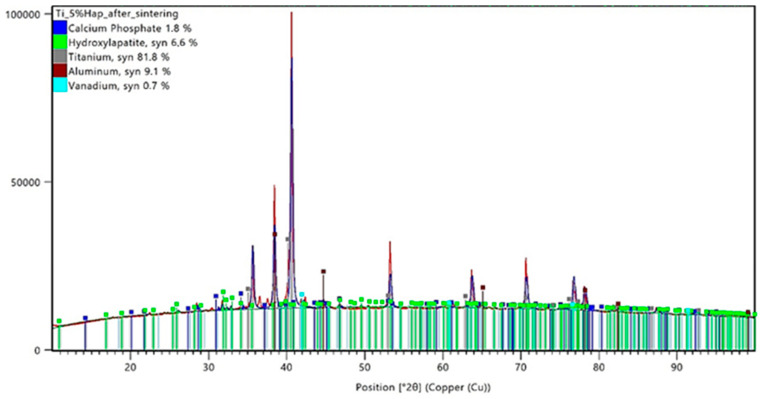
XRD analysis results for a sample containing 5% HAp after the sintering process.

**Figure 8:**
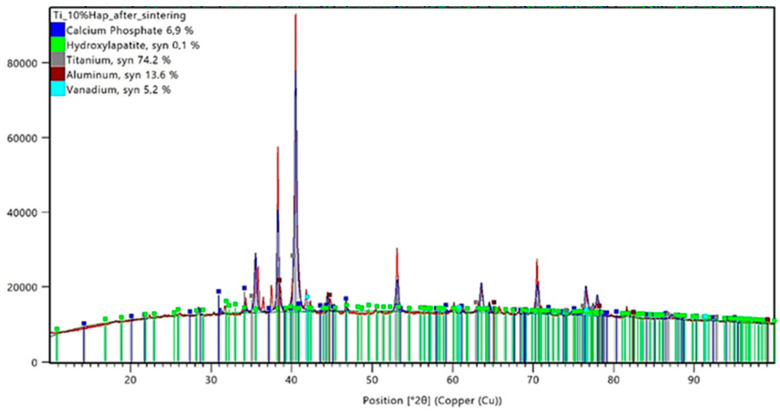
XRD analysis results for a sample containing 10% HAp after the sintering process.

The conducted X-ray diffraction (XRD) analysis aimed to evaluate the impact of the sintering process on the phase composition of titanium–hydroxyapatite (Ti-HAp) composites containing 5% and 10% HAp. Prior to sintering, the samples consisted of a mixture of the Ti-6Al-4V alloy and hydroxyapatite, with 5% carboxymethylcellulose (Car) used as a porogen. The XRD analysis results for the samples before sintering are presented in Figure 5 and Figure 6, while those for the sintered samples are shown in Figure 7 and Figure 8.

The XRD analysis for the Ti-5%HAp sample before sintering revealed the presence of dominant phases, including titanium (75.6%), hydroxyapatite (9.7%), aluminum (14.5%), and vanadium (0.2%). The distinct diffraction peaks for HAp suggest that the ceramic phase was well dispersed within the titanium matrix. In the Ti-10%HAp sample before sintering, the hydroxyapatite content was higher at 11.2%, while titanium accounted for 81.8% of the material volume. The aluminum and vanadium contents in this sample were 6.3% and 0.7%, respectively.

A noticeable discrepancy exists between the nominal hydroxyapatite content in the material composition and the values obtained from the XRD analysis. In the Ti-5%HAp sample, the detected hydroxyapatite content was higher than expected at 9.7%, while in the Ti-10%HAp sample, the measured value was 11.2%. It is important to emphasize that the XRD analysis only examines the surface layer of the sample. Since HAp is a ceramic phase with a different morphology than titanium, the preferential surface distribution of hydroxyapatite may occur. Additionally, the porous microstructure resulting from the presence of carboxymethylcellulose (Car) as a porogen may also influence the results. During the sample preparation, the local densification of HAp in more porous regions could have taken place, further affecting the XRD measurements. 

Following the sintering process at 1200 °C, the phase composition of the composites underwent significant changes. In the Ti-5%HAp sample after sintering, the hydroxyapatite content decreased to 6.6%, indicating a partial degradation of this phase. Additionally, new diffraction peaks corresponding to calcium phosphate (Ca_3_(PO_4_)_2_) appeared, with its content reaching 1.8%. Simultaneously, the relative content of titanium increased to 81.8%, which may be attributed to the reduction of the ceramic phase and the decrease in the aluminum (9.1%) and vanadium (0.7%) content.

In the Ti-10%HAp sample after sintering, an almost complete degradation of hydroxyapatite was observed, with its content dropping to only 0.1%. At the same time, the calcium phosphate phase significantly increased to 6.9%, which clearly indicates the decomposition of HAp into TCP. The titanium content decreased to 74.2%, while the aluminum and vanadium content increased to 13.6% and 5.2%, respectively. The absence of detectable titanium oxide (TiO_2_) peaks in both sintered samples suggests that the applied protective method was effective in preventing titanium oxidation.

The analysis of the data presented in Table 6 clearly indicates phase transformations of hydroxyapatite (HAp) occurring under high-temperature sintering conditions. The detection of the tricalcium phosphate (Ca_3_(PO_4_)_2_, TCP) phase in both samples after sintering confirms the progressive degradation of HAp as a result of thermal dehydroxylation and dehydration processes. These mechanisms lead to the destabilization of the HAp crystal structure, promoting its transformation into thermally more stable phosphate phases. The extent of this transformation is directly correlated with the initial hydroxyapatite content—the higher the ceramic phase concentration, the more pronounced the decomposition, as evidenced by the increased presence of TCP after sintering. The progression of the HAp degradation is also determined by the degree of the dispersion of the ceramic phase within the metallic matrix. In samples with a lower HAp content, the ceramic particles are more uniformly distributed, which limits the reactive contact surface and reduces the efficiency of the mass and heat transport, thereby slowing down the degradation process. In contrast, systems with higher HAp concentrations exhibit a more pronounced agglomeration of the ceramic phase and intensified local degradation—which shifts the reaction equilibrium toward TCP formation. Consequently, a higher initial HAp content does not enhance the thermal stability of the ceramic phase but rather promotes its breakdown under high-temperature sintering conditions.

### 3.5. SEM Analysis—SPS Samples

**Figure 9:**
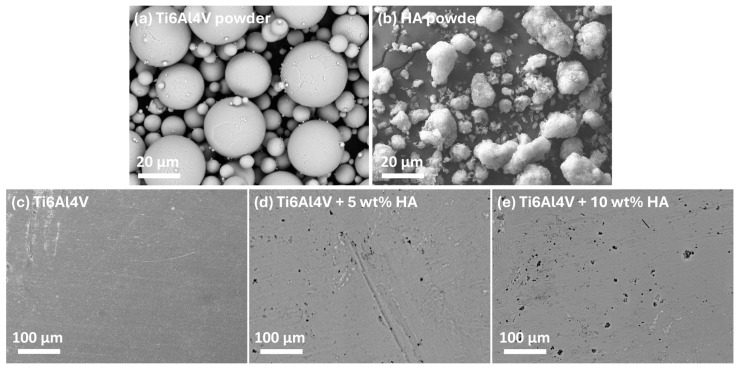
SEM images of SPS feedstock and consolidated bulks: (**a**) Ti alloy powder; (**b**) HAp powder; (**c**) SPS Ti alloy; (**d**) SPS Ti alloy with 5 wt% HAp; and (**e**) SPS Ti alloy with 10 wt% HAp.

Figure 9 presents SEM images of the feedstock powders used for SPS and the resulting consolidated Ti-HAp bulks. The Ti alloy powders exhibit a predominantly spherical morphology, whereas the HAp powder displays an irregular, spongy structure. The selection of primarily spherical powders for SPS is critical, as their geometry significantly enhances the packing density, leading to an improved initial compaction and reduced porosity in the final composite [58]. This optimized packing arrangement also facilitates efficient particle rearrangement during sintering, promoting a uniform densification. Furthermore, the uniform shape minimizes stress concentration points, thereby enhancing the mechanical properties of the composite. The superior interparticle contact achieved in SPS, coupled with ultra-fast heating and short dwell times, effectively suppresses grain growth and phase decomposition, resulting in a refined microstructure with a superior mechanical integrity [59].

Table 7 presents the relative density, porosity, and hardness (HV10 and HV0.2) of the bulks sintered by SPS. The SPS-consolidated samples exhibit a uniform distribution of the HAp phase within the Ti matrix. The bulks achieved high relative densities of approximately 96%, 98%, and 99% for the pure Ti alloy, 5 wt% HAp, and 10 wt% HAp, respectively. Correspondingly, the porosity of the SPS bulks remained below 2%, 6%, and 10% for the Ti alloy, 5 wt% HAp, and 10 wt% HAp, respectively. Although the incorporation of HAp led to a slight increase in porosity, the relative density remained high, demonstrating the material’s overall densification efficiency.

Regarding pore size, the 5 wt% HAp composite exhibited pores smaller than 10 µm. However, with an increase in HAp content to 10 wt%, the average pore size increased to approximately 15 µm, with some occasional pores reaching around 20 µm. The EDS analysis confirmed the widespread presence of the Ti metal phase, with significant amounts of Ti, Ca, and P concentrated within the pores. It is possible that pores originated from some degree of HAp agglomeration, as stated by Veljović et al. [60]. This indicates that the pores represent regions enriched with metal–ceramic phases. The observed trend of increasing porosity and pore sizes with higher HAp contents aligns with findings reported in the literature [61,62].

In terms of hardness, SPS samples exhibited comparable macro- and micro-hardness values for the pure Ti alloy and the 5 wt% HAp composite, measuring between 495 and 504 HV10 and 270–290 HV0.2, with the pure Ti alloy demonstrating slightly higher values. However, with the addition of 10 wt% HAp, the hardness decreased to 470 HV10 and 232 HV0.2. Overall, the hardness measurements obtained through the SPS process correlate well with the observed structural changes and relative density values, confirming the relationship between the composition, porosity, and hardness.

### 3.6. Results from LASER Texturing

**Figure 10:**
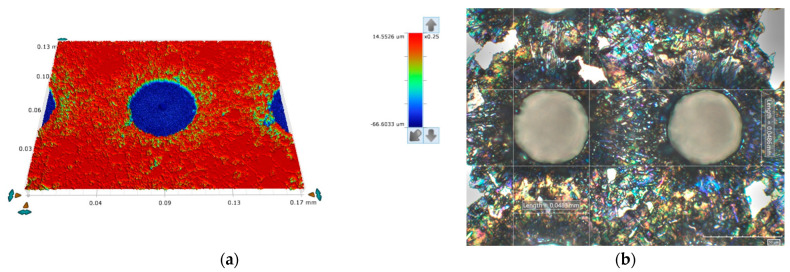
Textured sample of pure Ti6Al4V alloy recorded with optical interferometer (**a**) and digital microscope (**b**).

The surface of the reference sample (Ti6Al4V alloy), shown in Figure 10, was laser-textured using the drilling technique, creating holes with a mean diameter of 48.5 ± 0.2 μm and a depth of 74.8 ± 0.2 μm. The laser parameters were chosen to minimize the heat-affected zone and the recast layer of molten ejecta around the hole. A balance was achieved between the required high energy per pulse and the relatively short pulse duration (*E*_p_ = 380 μJ, *t*_p_ = 120 ns).

**Figure 11:**
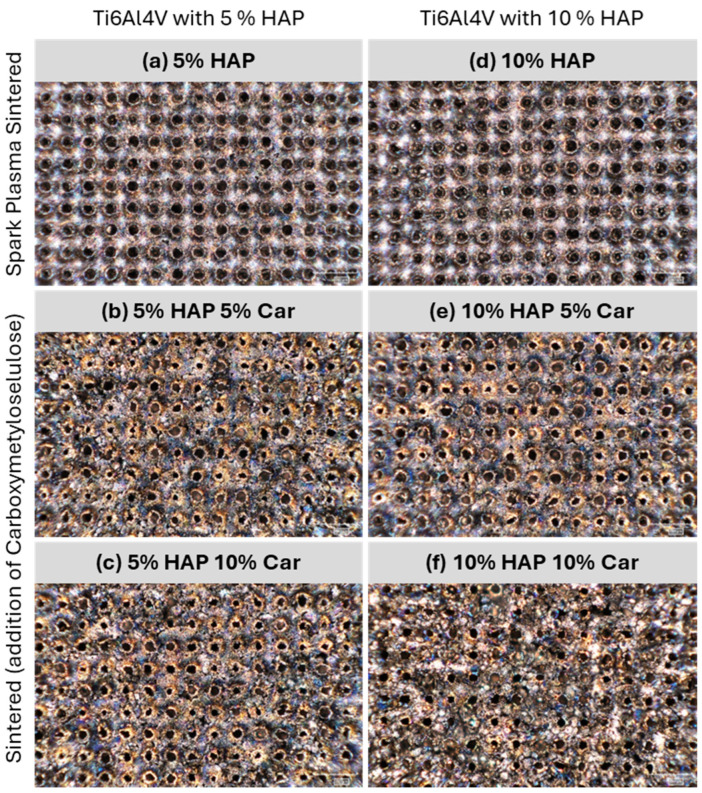
Optical images of studied Ti-HAP samples after laser texturing recorded by digital microscope.

Compared to the drilled holes on the reference sample and using the same laser parameters, the drilled hole dimensions on the studied Ti-HAP samples show a greater variance (see Figure 11), with the diameter generally larger and the depth shallower than the reference (see Table 8). This can be attributed to the heterogeneous optical properties of the composites, with the difference in the optical absorbance of the base material and the HAP being two orders of magnitude (*α*_Ti6Al4V_ ≈ 10^0^ cm^−1^, *α*_HAP_ ≈ 10^−2^ cm^−1^), possibly leading to a non-uniform material removal, and the porosity of the samples with Car as a result of the manufacturing process (sintering), where the Car evaporates due to a lower boiling point than the melting temperature of the base material (*T*_B, Car_ = 274 °C, *T*_M, Ti6Al4V_ = 1500–1670 °C), allows the molten and ablated material to escape in directions other than the irradiated area, resulting in the greater irregularity of the hole geometry.

### 3.7. Results from Tribological Tests

**Figure 12:**
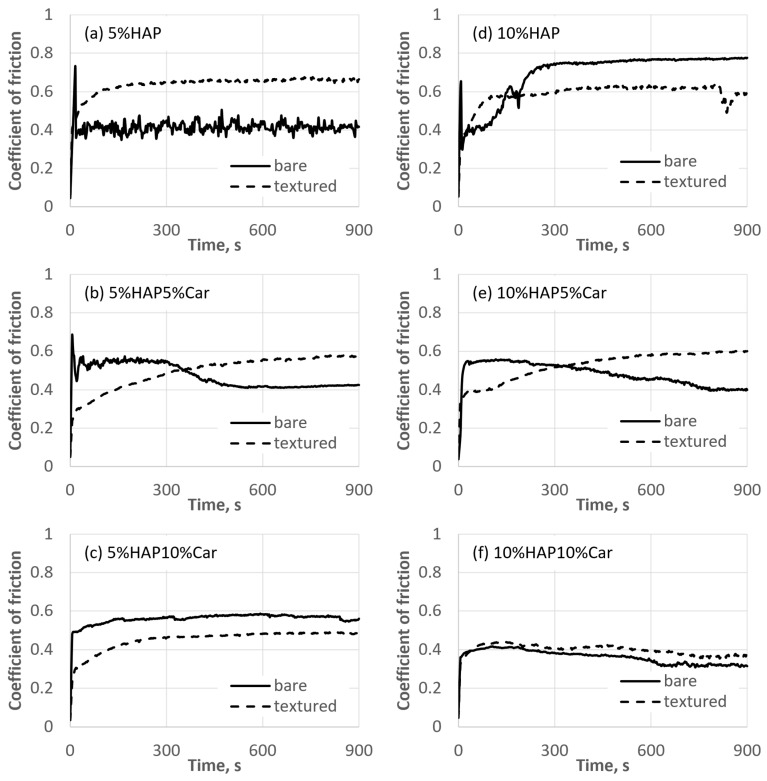
Evolutions of the coefficients of friction during tribological tests for Ti-HAP samples studied (the representative tests are shown and un-textured samples are added for comparison).

The representative evolutions of the coefficient of friction for each type of Ti-HAP sample during the tribological tests are reported in Figure 11. To reveal the effect of surface texturing on tribological performance, the results for the bare samples (without texturing) are added. Textured samples, in general, provided a higher, but also more stable, coefficient of friction with less fluctuations during the test. These differences in the fluctuations of the coefficient of friction between un-textured and textured samples are more significant for samples with 5% HAP (see Figure 12a–c), compared to samples with 10% HAP (see Figure 12d–f).

**Figure 13:**
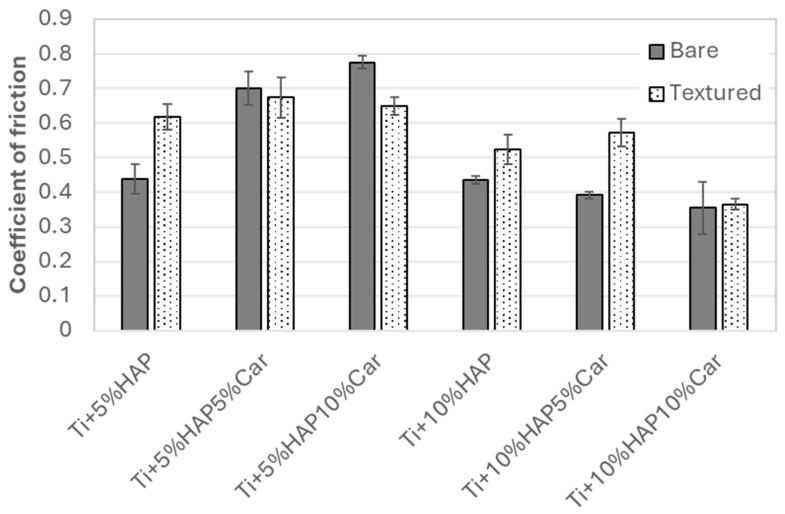
Steady-state coefficient of friction for Ti-HAP samples studied (un-textured samples are added for comparison).

Furthermore, the average steady-state coefficient of the friction results along with the standard deviation are reported in Figure 13. The texturing of the samples significantly increased the coefficient of friction compared to bare, un-textured, samples for both SPS samples without porosity (Ti + 5%HAP and Ti + 10%HAP). For all sintered samples with porosity, the coefficient of friction’s increase for textured samples was observed only for the Ti + 10%HAP5%Car sample. For samples Ti + 5%HAP5%Car and Ti + 10%HAP10%Car there were so significant changes, while for the Ti + 5%HAP10%Car even a decrease in the coefficient of friction was observed for the textured sample. The comparison between textured samples further reveals that samples with 5%HAP provide a higher steady-state coefficient of friction (between 0.62 and 0.67) compared to samples with 10%HAP (between 0.36 and 0.57), see Figure 13. The lowest steady-state coefficient of friction of 0.36 was achieved for the sample with the highest amount of HAP and the highest porosity (Ti + 10%HAP10%Car).

**Figure 14:**
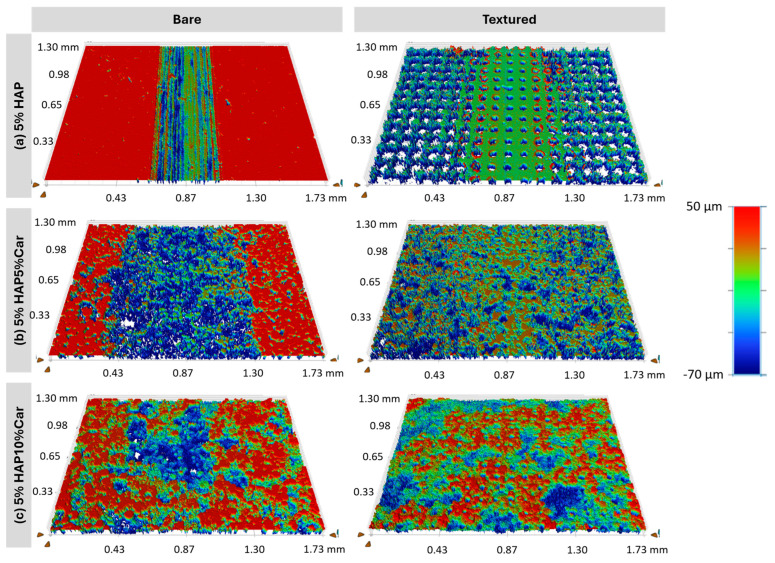
White-light interferometry images of bare and textured samples with 5%HAP.

**Figure 15:**
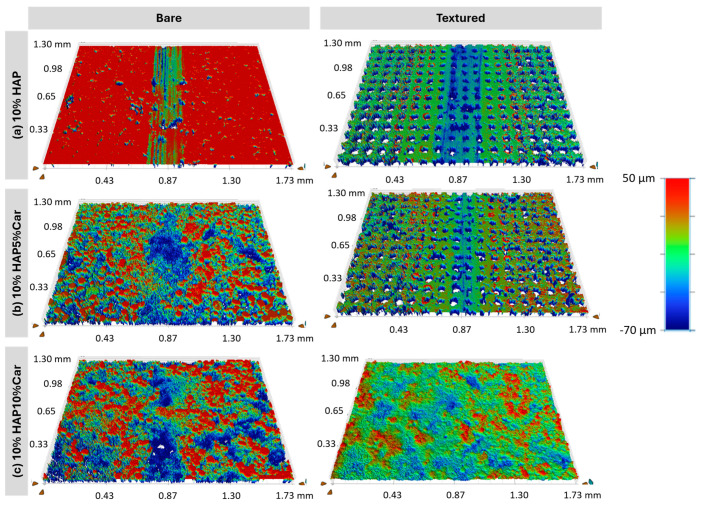
White-light interferometry images of bare and textured samples with 10%HAP.

White-light interferometry images of the Ti-HAP samples after the tribological tests are presented in Figure 14 and Figure 15, while the wear coefficients extracted from Figure 14 and Figure 15 are reported in Figure 15. Uniform wear tracks are visible for samples without (Ti + 5%HAP and Ti + 10% HAP) or with a low porosity (Ti + 5%HAP5%Car and Ti + 10%HAP5%Car), see Figure 14 and Figure 15. For both samples with the highest porosity (Ti + 5%HAP10%Car and Ti + 10%HAP10%Car) there is a less uniform wear track, but more areas with thorn materials.

**Figure 16:**
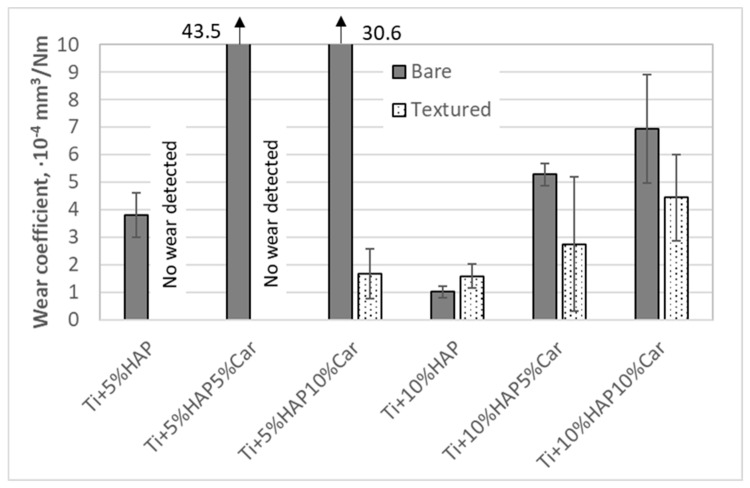
Wear coefficient of bare and textured Ti/HAP samples.

Wear coefficients from Figure 16 further show that a significant wear resistance improvement was achieved when the samples with 5%HAP were laser-textured. The improvement in the wear resistance was especially pronounced for the Ti + 5%HAP and Ti + 5%HAP5%Car samples, where no wear was even detected for both textured samples. On the other hand, the wear resistance improvement when texturing the samples with 10%HAP was not achieved (sample Ti + 10%HAP) or it was within the uncertainty of the measurement (samples Ti + 10%HAP5%Car and Ti + 10%HAP10%Car).

### 3.8. Incubation Studies

#### 3.8.1. Potentiometry Analysis

**Figure 17:**
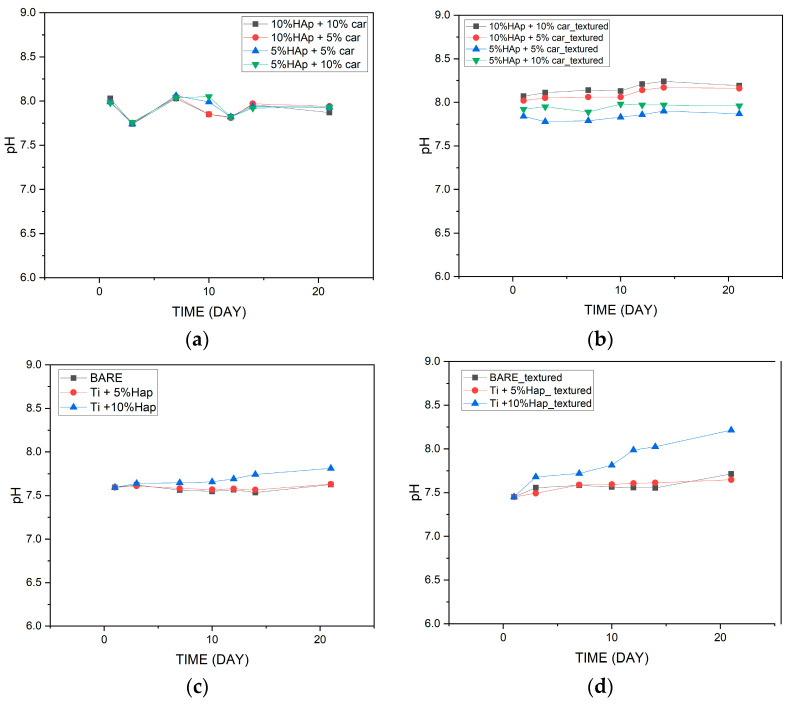
Measured pH values for powder metallurgy samples (**a**,**b**) and SPS samples (**c**,**d**) during incubation in SBF.

Potentiometric studies, including the observation of changes in pH values, make it possible to determine the stability of biomaterials in a suitable environment. During the incubation studies, pH values were measured over a period of 21 days. Two groups of materials, produced by two different methods—powder metallurgy and SPS, were subjected to incubation tests. Both groups of materials were measured before and after the laser structuring. The results are shown in Figure 17.

It was observed that the incubation of materials produced by the SPS method unstructured by a laser is characterized by slightly lower pH values during the whole incubation process. On the other hand, unstructured materials produced by the powder metallurgy method cause greater fluctuations in the pH values of the artificial saline fluid during incubation. However, in both cases, no sudden jumps in the pH values of the fluid were observed to indicate abnormalities, and the pH values oscillate relatively at the same level.

For the samples after the laser structuring, similar relationships are observed as before. However, for samples produced by the powder metallurgy method after laser structuring, smaller fluctuations in SBF pH values were observed throughout the incubation process.

It was found that the laser structuring process does not significantly affect the pH values of the incubation fluid during the incubation process. The increased amount of hydroxyapatite in the case of laser-structured materials causes higher pH values during the last days of incubation. This observation applies to materials produced by both methods

#### 3.8.2. Conductivity Analysis

**Figure 18:**
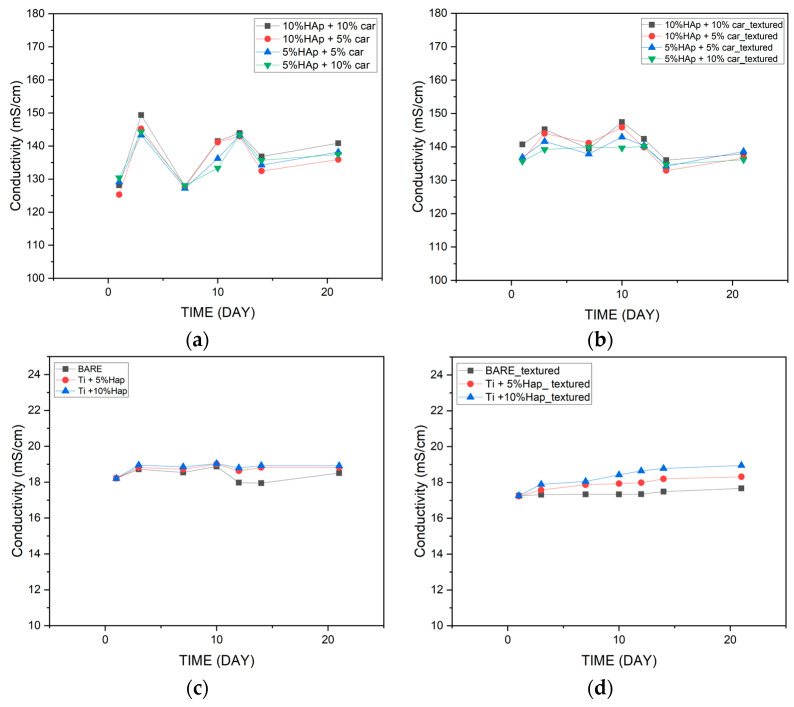
Measured conductivity values for powder metallurgy samples (**a**,**b**) and SPS samples (**c**,**d**) during incubation in SBF.

The conductometric analysis of the reacting compounds makes it possible to analyze ionic compounds and at the same time monitor chemical reactions through conductivity measurements. Electrolytic conductivity measurements were carried out in artificial plasma (SBF). The results are shown in Figure 18. Changes in conductivity indicate ion exchange between the material and the incubation fluid. Both materials before and after structuring behave in a similar manner. Materials produced by the SPS method show an ionic conductivity of about 18 mS/cm throughout the incubation period, regardless of the hydroxyapatite content. In the case of samples produced by the powder metallurgy method, there are greater fluctuations in the ionic conductivity of the incubation fluid, with values varying from 125 mS/cm to about 140 mS/cm. This difference may be due to the different ways in which the materials studied were produced. Higher ionic conductivity values for materials produced by the powder metallurgy method may indicate a more intense reaction of the material with the incubation fluid.

## 4. Discussion

Over the years, a great deal of research has been carried out on coating implants with hydroxyapatite, but this idea, despite its many advantages, also has drawbacks, such as its clinical limitations. HAp-coated implants in particular have found application in dental implants. This way of inserting hydroxyapatite can be characterized by an increased bacterial susceptibility compared to titanium implants. Such limitations were presented by Ong JL et al. in a publication [63]. In contrast, concerns regarding microbial susceptibility, resorption, fatigue, and fracture in long-term applications were presented in an article by Kiodo H et al. [64]. In contrast, from the article by Lee JJ et al., it can be deduced that hydroxyapatite-coated implants achieved similar results regarding the long-term survival of dental implants compared to uncoated ones [65]. Thus, the creation of the innovative composites presented in this article—the titanium–hydroxyapatite alloy—gives new light to this field of science and the prospects for better implants without the clinical limitations mentioned above.

In this study, the effects of the fabrication techniques of Spark Plasma Sintering (SPS) and powder metallurgy (PM) on the properties of laser-textured Ti64/HAp composites were investigated. The results show that the processing route significantly affects the microstructure, mechanical performance, and surface properties of the materials. SEM/EDX analyses have confirmed the signals from phosphorus and calcium, which confirms the presence of cauliflower-like visible HAp structures (Figure 1c). Moreover, the surface is ridged and strongly corrugated which makes it perfect for cell adhesion and growth, boosting osteoconductivity. The approach of comparing two different material fabrication methods and then using texturing to increase the specific surface area of the resulting composites is a highly innovative and forward-thinking solution. This two-phase methodology not only offers a novel pathway for optimizing surface properties but also has significant potential in the design of next-generation biomedical implants, especially those for demanding applications such as joint replacements.

The XRD analysis allows for the evaluation of the phase stability of hydroxyapatite (HAp) in the titanium composite under high-temperature sintering conditions. The results indicate that sintering at 1200 °C leads to HAp degradation, the extent of which depends on its initial content in the material. In the Ti-5%HAp sample, the partial decomposition of hydroxyapatite was observed, resulting in the formation of Ca_3_(PO_4_)_2_ (TCP). In contrast, in the Ti-10%HAp sample, the degradation was nearly complete, indicating that hydroxyapatite is not thermodynamically stable under these conditions. This phenomenon can be explained by the dehydroxylation and dehydration of hydroxyapatite, which lead to its gradual transformation into TCP at temperatures above 1000 °C. A significant observation from the results is that the degradation in the Ti-5%HAp sample was less pronounced compared to the Ti-10%HAp sample, where almost the entire hydroxyapatite phase transformed into TCP. This effect may be attributed to the fact that a higher HAp content increases the local concentration of the ceramic phase, facilitating degradation mechanisms. Hydroxyapatite is thermally less stable than titanium, and its greater presence promotes factors leading to decomposition, such as dehydroxylation and dehydration, which may occur more intensively. In the Ti-5%HAp sample, titanium constitutes a larger portion of the material, potentially stabilizing the microstructure and slowing down the HAp decomposition by limiting the direct interaction of hydroxyapatite particles. HAp degradation may also be associated with diffusion effects and interfacial contact. In the Ti-10%HAp sample, HAp particles have a greater tendency to aggregate, increasing the reaction surface area and the likelihood of the transformation into TCP. Conversely, in the sample with a lower HAp content, individual HAp particles are more dispersed within the titanium matrix, which may delay degradation by physically separating the phases. Other key factors are heat distribution and water vapor transport, which result from both the dehydroxylation and dehydration of HAp. At lower temperatures (below 500 °C), dehydration occurs, involving the removal of adsorbed and intercrystalline water, whereas at higher temperatures (above 850–1000 °C), the dominant mechanism is dehydroxylation, leading to the loss of OH^−^ groups and the formation of intermediate phases. In the Ti-10%HAp sample, a higher HAp content results in a greater amount of released water, both in the form of physically bound water (dehydration) and that originating from OH^−^ breakdown (dehydroxylation). This may locally accelerate the transformation into TCP by shifting the equilibrium of these reactions. In contrast, in the Ti-5%HAp sample, the lower amount of released water may slow down the decomposition rate of HAp.

The XRD analysis results align with the findings from incubation studies in simulated body fluids, confirming the influence of phase transformations during the sintering of the chemical properties of Ti-HAp composites. In the Ti-5%HAp sample, where a significant portion of hydroxyapatite remained intact after sintering, pH values remained stable during incubation, suggesting a higher resistance to dissolution and better ionic balance control in the solution. In contrast, in the Ti-10%HAp sample, where HAp completely degraded into TCP, a strong alkalization of the solution was observed. This effect is attributed to the higher solubility of TCP in aqueous environments, leading to the intensified release of calcium (Ca^2^^+^) and phosphate (PO_4_^3−^) ions. Additionally, under alkaline conditions, a reaction between Ca^2^^+^ ions and OH^−^ may occur, resulting in the formation of calcium hydroxide (Ca(OH)_2_), which further stabilizes the high pH of the solution.

The tribological behavior of Ti-HAP samples can be elaborated first by the effect of the sample production method and the porosity (% of Car) on the result of the laser surface texturing, and the further joint effect of the production method and porosity on the amount of HAP and surface laser texturing on the tribological performance. The most uniform dimples produced by the laser texturing were achieved for both SPS Ti-HAP samples (5%HAP and 10%HAP), signifying the superior texturing ability of SPS bulks, Figure 11a,d. For all samples with porosity (addition of Car in 5% or 10%) the dimples were much less uniform, irregularly shaped, and of different dimensions (see Figure 10b,c,e,f). For recent studies of the LST Ti alloy this was not the case, since the samples were not produced in the way that obtains a controlled ‘build-in’ porosity by the addition of Car []. For this reason, this feature cannot be neglected when discussing the observed tribological performance of the Ti-HAP samples studied. After texturing, the COF, in general, was higher but much more stable for the 5%HAP sample, and the wear was also much more improved for the 5%HAP sample, and so the porosities increase the wear. The texturing helps only with the 5%HAP sample.

Incubation studies show us the application potential of materials produced by both methods. The structured laser process does not significantly affect pH values and conductivity. After the entire incubation period, it was found that materials with the smallest amount of hydroxyapatite showed the smallest fluctuations in the parameters studied, regardless of the manufacturing method. Based on the incubation tests carried out, it can be concluded that the best properties that can affect the application of materials in a living organism are shown by materials with 5% hydroxyapatite.

The fabricated biomaterials by both methods, i.e., powder metallurgy and SPS, show a strong application potential in the medical field. The addition of hydroxyapatite and carboxymethylcellulose, which affects porosity, strongly influences the osteointegration potential. This simultaneously confirms the utility of these biomaterials for bone regeneration. The presented results suggest potential applications of titanium–ceramic composites in the field of medicine and implantology. Our proposed solution, both produced by powder metallurgy and the SPS method, has the potential to be used as implants for bone defects and especially for uses as a joint prosthesis.

## 5. Conclusions

This article presents a comparison of two methods of manufacturing metallic–ceramic composites and then compares their physicochemical properties and performs tribological tests. It was found that both types of materials were successively produced by both methods, i.e., powder metallurgy (PM) and Spark Plasma Sintering (SPS).

In the case of samples made by the powder metallurgy method, the XRD analysis showed that some of the hydroxyapatite degraded, resulting in the formation of TCP. A greater ceramic degradation was observed for samples with higher hydroxyapatite contents. This phenomenon indicates the instability of HAp at the applied temperature of 1200°C, leading to the gradual transformation of hydroxyapatite. Further studies should consider using a lower sintering temperature and repeating the analyses to help determine the stability of the ceramics.

Tribological tests helped to evaluate the wear of the material depending on the manufacturing method, but also on the content of the bioactive ceramics and porophore. It was found that more uniform pits were formed for samples produced by the SPS method, and samples produced by powder metallurgy were heterogeneous. The texturing process caused an increase in the COF. It was found that as the porosity increased, the wear increased.

Incubation studies conducted in SBF indicated the application potential in the living organism of both types of materials. Materials with 5% bioactive ceramics showed the most stable results.

The presented research demonstrates the biomedical application potential of Ti/HAp composites produced by various methods. Further research should be focused on biological studies that will evaluate the cytotoxicity of the materials and at the same time assess the applicability of the materials in a living organism.

## Figures and Tables

**Table 1:** Elemental composition of Ti6Al4V powder.

Elements/Composition	Ti	Al	V	Fe	O	N	H
Ti6Al4V wt. %	Bal	5.5–6.5	3.5–4.5	0–0.25	0–0.13	0–0.05	0–0.012

**Table 2:** Powder metallurgy sample composition.

	Sample	Titanium Alloy	Hydroxyapatite [Hap]	Carboxymethylcellulose [CMC]
1	10%Hap 10%Car	Ti6Al4V	10%	10%
2	10%Hap 5%Car	Ti6Al4V	10%	5%
3	5%Hap 10%Car	Ti6Al4V	5%	10%
4	5%Hap 5%Car	Ti6Al4V	5%	5%

**Table 3:** SPS sample composition (wt.%).

	Sample	Titanium Alloy	Hydroxyapatite [Hap]
1	BARE	Ti6Al4V	-
2	Ti + 5%Hap	Ti6Al4V	5%
3	Ti + 10%Hap	Ti6Al4V	10%

**Table 4:** The EDX analyses of the visible surface of Figure 1c.

Element Symbol	Atomic Concentration [%]	Weight Concentration [%]
C, Carbon	26.78	8.70
Al, Aluminum	1.23	0.90
P, Phosphorus	6.44	5.40
Ca, Calcium	0.92	1.00
Ti, Titanium	61.43	79.60
V, Vanadium	3.19	4.40

**Table 5:** The porosity and density of the samples.

Samples	Porosity [%]	±SD [%]	Density [g/cm^3^]	±SD [g/cm^3^]
10%HAP 10%Car	25.856	0.148	2.783	0.0485
5%HAP 10%Car	22.244	1.109	2.994	0.089
10%HAP 5%caCar	14.573	0.637	3.287	0.086
5%HAP 5%Car	15.693	0.519	3.149	0.144

**Table 6:** Comparison of the phase composition of Ti-HAp composites before and after sintering.

Sample	Titanium, %	Aluminum, %	Vanadium, %	HAp, %	TCP, %
Ti_5%HAp_before sintering	75.6	14.5	0.2	9.7	-
Ti_10%HAp_before sintering	81.8	6.30	0.7	11.2	-
Ti_5%HAp_after sintering	81.8	9.10	0.7	6.6	1.8
Ti_10%HAp_after sintering	74.2	13.6	5.2	0.1	6.9

**Table 7:** Relative density, porosity, and hardness of Spark Plasma sintered bulks.

Materials	Relative Density (%)	Porosity (%)	Hardness	
			HV 0.2	HV 10
Ti64 + 5 wt% HA	98.4	<6	495 ± 10	270 ± 06
Ti64 + 10 wt%HA	96.2	<10	470 ± 15	232 ± 30
Ti64	99.9	<2	504 ± 10	293 ± 10

**Table 8:** Measured geometrical properties of dimples on studied Ti-HAP samples.

Sample	Dimple Depth, µm	Dimple Diameter, µm	Raster, µm
Reference Ti6Al4V	74.6–75.0	48.3–48.6	100
Ti + 5%HAP	63.0–67.3	51.2–52.8	100
Ti + 5%HAP + 5%Car	74.5–76.9	45.2–50.0	100
Ti + 5%HAP + 10%Car	66.0–66.5	45.5–47.1	100
Ti + 10%HAP	62.1–63.7	52.3–55.9	100
Ti + 10%HAP + 5%Car	62.1–64.2	54.0–54.8	100
Ti + 10%HAP + 10%Car	61.2–63.4	48.3–49.1	100

## Data Availability

The original contributions presented in this study are included in the article, further inquiries can be directed to the corresponding authors.
